# Supplementary material for: Sustained neural rhythms reveal endogenous oscillations supporting speech perception
Source: PLoS Biol. 2021 Feb 26;19(2):e3001142. doi: 10.1371/journal.pbio.3001142 (PMC7946281; doi:10.1371/journal.pbio.3001142)
Supplement: Attachment — Submitted filename: rebuttal_FINAL.pdf [file pbio.3001142.s007.pdf]

Reviewer #1

The study by van Bree et al. investigated whether endogenous neural oscillations played a role in speech perception and whether they were related to the neural responses entrained to speech. The authors found that (1) the neural responses to rhythmic speech stimuli could last for ~1-2 seconds after the stimulus offset and (2) the phase of neural response to speech could predict how the tACS phase modulated speech perception. Based on these two findings, the authors concluded that the neural responses to speech were related to spontaneous neural oscillations.

In general, the study addressed an important question regarding to the neural mechanisms underlying speech perception. The methods were sophisticated and well described. The tACS results were quite interesting. Nevertheless, I have some concerns about whether the findings can indeed support the conclusion.

We thank the Reviewer for interesting and helpful comments and hope that we were able to address remaining concerns.

1) First, the authors show some evidence that the entrained neural response could last for 1-2 seconds after the stimulus. Nevertheless, as the authors pointed out, the entrained response and the sustained response are generated from different neural sources, suggesting that the entrained response is not "endogenous neural oscillations aligned (or "entrained") to the stimulus rhythm".

We thank the Reviewer for this interesting point. Indeed, we show that rhythmic MEG responses observed *during* rhythmic sensory stimulation are unlikely to stem from identical sources as compared to those measured *after* stimulus offset. However, we believe that such a finding does not speak against the sustained oscillations being an “echo” from endogenous oscillations aligned to rhythmic speech. This is because we hypothesize the entrained response to include both evoked responses and endogenous oscillations, with the former dominating the response (described in detail in our response to point 6 below). A change in the estimated neural source is therefore to be expected after stimulus offset, when evoked responses are absent but oscillations persist. Such a change, however, does not suggest that sensors/sources capturing sustained oscillations are *inactive* in the entrained time window – they might simply not be the strongest factor driving the response. Indeed, sensors capturing sustained oscillations also show a significantly entrained response during sensory stimulation (Fig. 3C, red; previously Fig. 2J). This point is now clarified in the revised manuscript (p. 22 in version with tracked changes):

“Based on the observation of sustained oscillatory responses after stimulus offset, we conclude that an endogenous oscillatory system is involved in such entrained brain responses. Although endogenous oscillations are difficult to measure during stimulation, the most parsimonious explanation of our results is that the entrained response entails both evoked responses and endogenous oscillations, with the former dominating the response. After stimulus offset only the latter prevails, leading to a change in topographical pattern and estimated source. Indeed, we found that sensors capturing sustained oscillations also show a significantly entrained response during sensory stimulation (Fig. 3C, red), while stronger, stimulus-driven activity at distinct sensors, quickly subsided after stimulation (green in Fig. 3C).”

We furthermore note that a similar shift towards parietal sensors after rhythmic stimulation has been observed in another recent study (Bouwer et al. 2020, *BioRxiv*).

2) Second, the sustained response does not correlate with behaviour while the entrained response does, providing further evidence that the sustained response does not directly contribute to speech perception.

We agree with the Reviewer that the absence of a correlation with behaviour might, at first glance, speak against an important role for the observed response. There are, however, a number of points we would like to clarify. First, we did not collect a behavioural measure of speech perception success in Experiment 1. Rather, we measured the ability of listeners to detect a temporal irregularity, a task that

could be performed on an unintelligible control stimulus (1-channel vocoded speech) as well as on intelligible speech (16-channel) and hence allowed us to test for speech-specific effects. In previous work (Zoefel et al. 2018, *Curr Biol*), we demonstrated speech-specific effects of tACS for fMRI responses during the same task. Testing the success of speech perception in one (16-channel speech), but not the other condition (1-channel speech) would have made similar differences between conditions difficult or impossible to interpret.

Second, we explicitly avoided a task for which sustained neural activity is directly required for task performance. This could have involved target presentation during the silent period (i.e. during the hypothesized sustained activity), or a task that encouraged participants to imagine or tap along with the rhythm (e.g., if asked to predict upcoming stimuli). Such tasks might again have produced rhythmic neural activity, but this would not have reflected endogenous neural oscillations, but rather rhythmic processes that are inherent to the task (e.g., produced by participants tapping along with stimuli). Instead, to keep participants alert and attentive to the stimulus rhythm, we asked them to detect an irregularity in the rhythmic sounds. However, as this irregularity could only occur during sensory stimulation, it is not surprising that performance in this task is only correlated with neural activity which was measured at that time. In the revised manuscript, we tried to make this point clearer by explaining our rationale in more detail (p. 9/10), copied below. We also moved figures showing behavioural results from Experiment 1 to Supporting Information, to make it clearer that these analyses were not designed to measure behavioural relevance of the sustained effect.

"We did not measure the success of speech perception in Experiment 1. This is because such a task would have biased participants to attend differently to stimuli in intelligible conditions, making comparisons with neural responses in our unintelligible control condition difficult. Similarly, we refrained from using tasks which might have biased our measurement of endogenous oscillations in the silent period. For example, tasks in which participants are asked to explicitly predict an upcoming stimulus might have encouraged them to imagine or tap along with the rhythm. Our irregularity detection task was therefore primarily designed to ensure that participants remain alert and focused and not to provide behavioural relevance of our hypothesized sustained neural effect. Nevertheless, we correlated the RSR in both time windows (and at the selected sensors) with performance in the irregularity detection task (Fig. S2). […]"

3) Third, I have some doubts about whether the MEG sustained response indeed exist. First of all, it is a very weak response, not observable in the raw ITC spectrogram. Even in Fig. 2H, the 2 and 3 Hz ITC peaks are not the most prominent peaks in the relatively narrow frequency range being shown.

We appreciate the Reviewer's comment, but insist that our analysis pipeline was designed to reveal statistically robust effects while avoiding potential pitfalls, as explained in the following.
Like other spectral measures, ITC is affected by aperiodic ("1/f") activity, leading to larger ITC for lower frequencies without necessarily involving endogenous oscillatory activity. Such an effect can, for example, be seen in Fig. 3B (previously Fig. 2H; note the 1/f component in the panel "original"). Moreover, various neural responses can bias ITC, such as that to the omission of an expected stimulus. Such a response is visible for both stimulus rates in Fig. 1E, leading to an increase in ITC at most frequencies (strongest at lower frequencies). We therefore designed an analysis pipeline which controls for these issues. Most importantly, it contrasts ITC at a given neural frequency during stimulation at a corresponding rate (e.g., 2-Hz ITC during 2-Hz speech), with that at the same neural frequency but measured during stimulation with a different rate (e.g., 2-Hz ITC during 3-Hz speech). Consequently, generic processes that are independent of stimulus rate but might affect ITC, such as an omission response, are removed. In other words, in contrast to ITC, this measure reveals a rhythmic response that is specific to stimulus rate – therefore it was termed rate-specific response index (RSR). We tried to make this clearer in the revised manuscript (p. 6):

"Spectral measures such as ITC can be biased by other neural activity than endogenous oscillations: For example, a response caused by the omission of an expected stimulus might produce an increase in ITC that is most pronounced at low frequencies (~250 ms in Fig. 1E). By contrasting ITC between two

rate conditions, RSR removes such contamination if it is independent of stimulus rate (i.e. present in both rate conditions)."

We would like to bring two additional points to the Reviewer's attention: First, we now test for rate-specific responses for the two rate conditions separately (in addition to the RSR analysis that combines the two rates), providing further evidence that the observed rhythmic response is rate specific. Second, removing 1/f components in Fig. 3B does reveal peaks in the spectrum that closely correspond to the respective stimulus rates. Imperfect sinusoidal signals often produce harmonics in spectral analyses; other peaks in the spectrum might reflect such harmonics. All of this is now mentioned in the revised manuscript (p. 8/9 and caption of Figure 3):

"We first verified that the rate-specific responses, revealed in our main analyses, were produced by responses at both of the stimulus rates tested. We found this to be the case in both entrained (Fig. 3A) and sustained (Fig. 3B) time windows: ITC at both 2 Hz and 3 Hz was significantly higher when it corresponded to the stimulation rate than when it did not (entrained: 2 Hz, $t(20) = 13.11$, $p < 0.0001$; 3 Hz, $t(20) = 11.46$, $p < 0.0001$; sustained: 2 Hz, $t(20) = 1.91$, $p = 0.035$; 3 Hz, $t(20) = 2.17$, $p = 0.02$). In the sustained time window, subtracting 1/f components (dashed lines in Fig. 3B) from the data (continuous lines) revealed clearer peaks that correspond to the stimulation rate (or its harmonics). We note again the RSR discards such 1/f components by contrasting ITC values at the same two frequencies across the two stimulus rates."

[…]

"Note that the peaks correspond closely to the respective stimulus rates, or their harmonics (potentially produced by imperfect sinusoidal signals)."

An alternative to our approach would be to test significance of the "raw" ITC values, following the Reviewer's concern. However, ITC values are always larger than (or equal to) 0; the null distribution of these values therefore needs to be simulated for statistical evaluation, for example using permutation tests. The surrogate distribution, constructed for such tests, is assumed to include all properties of the original data *except of* the hypothesized rhythmic response. However, this assumption does not necessarily hold in our dataset: Randomising trials or conditions might also abolish increases in ITC due to omission response or 1/f components, and lead to false positive effects. To test whether this is the case, we constructed such a surrogate distribution by adding a random value to the phase extracted for each trial before re-calculating ITC values as described in the original manuscript. As shown below, ITC in the surrogate distribution is indeed not affected by the 1/f component that is visible in the original data. An appropriate null distribution would however contain such a component.

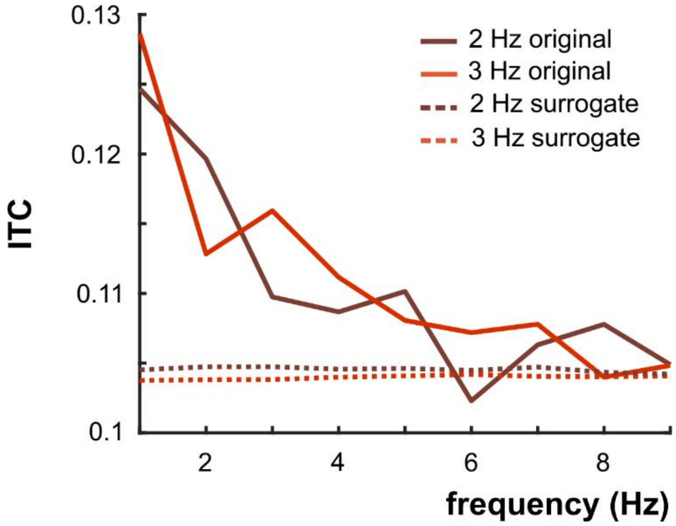

Figure R1. ITC as a function of neural frequency in the sustained time window, in response to the two stimulus rates (reproducing Fig. 3B), and for a surrogate dataset (mean across permutations; dashed lines). The latter was constructed by adding a value to the phase in each trial before calculating ITC. This procedure should abolish any phase consistency across trials and therefore simulate the null hypothesis of no rhythmic sustained response; however, it also abolishes the 1/f component visible in the original dataset.

We therefore believe that a comparison between two conditions (i.e. two stimulus rates) – i.e. the RSR – leads to statistically more robust results than corresponding permutation tests (p. 6):

"By contrasting ITC between two rate conditions, RSR removes such contamination if it is independent of stimulus rate (i.e. present in both rate conditions). This property makes it – in the present case – also superior to other commonly used approaches, such as permutation tests [21,22], which would not only abolish the hypothesized rhythmic responses, but also non-rhythmic responses which produce high ITC for other reasons (e.g., evoked response to stimulus omission)."

Finally, we ran additional verifications/analyses to demonstrate that the RSR is an appropriate measure: First, we verified that it is normally distributed. Second, we used the surrogate distribution, described above, to verify that the null hypothesis indeed corresponds to an RSR of 0. We found that the 95% confidence interval is centred on 0 and included this result in the revised manuscript (p. 35):

"For all sensors and conditions (intelligibility, duration) separately, we verified that the RSR is normally distributed ($p > 0.05$ in Kolmogorov-Smirnov test), a pre-requisite for subjecting it to parametric statistical tests. Note that such a behaviour is expected, given the central limit theorem (combining multiple measures leads to a variable that tends to be normally distributed). Fig. S5A shows the distribution of RSR, averaged across sensors and conditions. Finally, we constructed a surrogate distribution to verify that an RSR of 0 indeed corresponds to our null hypothesis. This was done by adding a random value to the phase in each trial before re-calculating ITC and RSR as described above, and repeating the procedure 100 times to obtain a simulated distribution of RSR values in the absence of a rhythmic response. This distribution of RSR values was indeed centred on 0, and its 95% confidence interval included 0 (Fig. S5B). Once again, this justifies our use of parametric statistical tests to confirm whether the observed RSR is greater than zero."

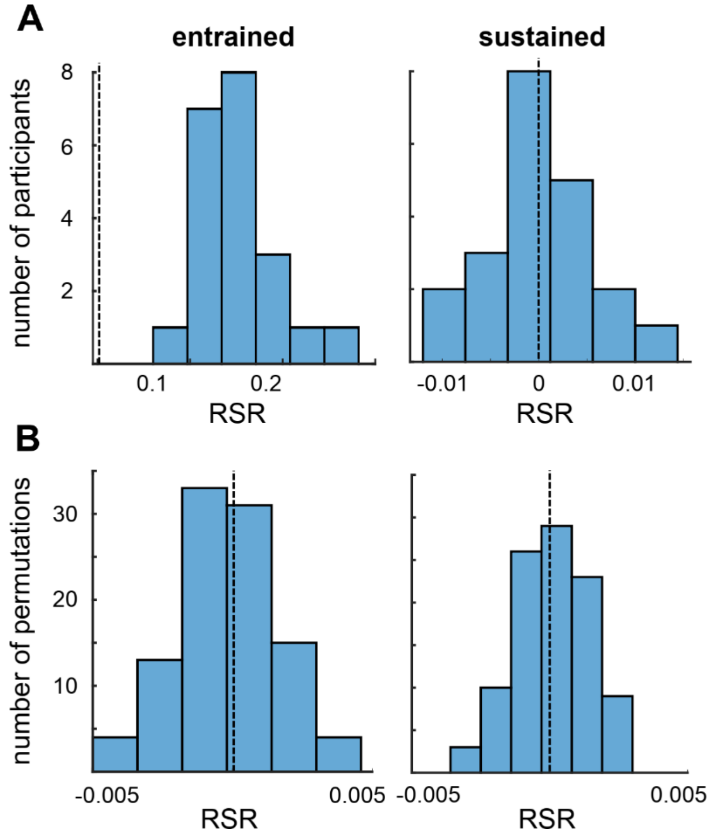

Figure S5. ***Control analyses validating RSR as an appropriate measure to reveal rate-specific rhythmic brain responses. A****. Distribution of RSR over participants. Note the approximate normal distribution as required for parametric tests (e.g., t-test against 0).* ***B****. Distribution of RSR, averaged across participants, in a surrogate dataset (see Materials and Methods). RSR is centred on 0 (dashed lines), validating our null hypothesis of RSR = 0. For all results shown here, RSR values have been averaged across sensors and conditions (corresponding to the average RSR shown in Fig. 2A,D), including those for which the RSR is not reliably different from 0. Statistically significant rate-specific responses after intelligible speech are shown in Fig. 2F. Note that x-axes are not identical across panels.*

Together, our approach consists of appropriate analytical steps to remove potential bias or contamination, and its outcome is a cleaner measure of whether sustained responses indeed exist.

4) Even if the sustained response is statistically significant, it is way smaller than the entrained response. Based on Fig. 2D and Fig. 2Hz, the ITC for entrained response is >0.2 while the ITC for sustained response is <0.12, with the chance-level ITC being above 0.1.

We would like to insist that large differences in ITC between entrained and sustained responses are expected, but this does not necessarily mean that the latter are absent or uninformative. Participants listened to sound sequences, presented at a comfortable volume; these sounds therefore produce strong evoked responses which will also be visible in the ITC, due to their regularity. As these sound-evoked responses were absent when sustained responses were measured, it is not surprising that we see a reduction in ITC. Consequently, the magnitude of entrained and sustained responses cannot be compared directly. Another important point is that we use sustained responses as evidence of the operation of another process that is distinct from these evoked responses: This neural "echo" arises from entrained endogenous oscillations, which we and others had hypothesized to exist, but which cannot be conclusively measured during sensory stimulation. A weaker (but significant) "echo" does not necessarily mean that this process of interest is weak as well, only that this effect is smaller than the evoked responses which have been observed for many decades.

We believe that the Reviewer may have mistaken the dashed lines in Fig. 3B (previously Fig. 2H) as significance threshold (they reflect a 1/f curve that was subtracted from the data for the rightmost panel), and we apologise for the lack of clarity. We now describe in the main text (p. 9):

"In the sustained time window, subtracting 1/f components (dashed lines in Fig. 3B) from the data (continuous lines) revealed clearer peaks that correspond to the stimulation rate (or its harmonics)."

Significance for ITC values can be tested using permutation procedures. However, as explained and illustrated (Fig. R1) in detail above, such a procedure can - in our case - lead to false positive results.

5) Furthermore, the sustained response is not observed for EEG.

We agree with the Reviewer that a demonstration of sustained rhythmic responses in the EEG would provide additional evidence for endogenous oscillations involved in speech processing. However, we do not believe that the absence of such an effect in the EEG speaks against the existence of sustained oscillations in general. First, MEG has a higher signal to noise ratio than EEG and might be more capable of measuring a neural signal that is not directly driven by external input (i.e. endogenous oscillations) and therefore of reduced magnitude (see previous point). Second, the neural signals captured with MEG and EEG differ in the location and orientation of the underlying neural sources. It is therefore possible that certain neural processes can be measured more successfully with one than the other of those methods. We insist on these points in the revised manuscript (p. 5 and 14):

"Due to its higher signal-noise ratio, we focused our initial analyses on the MEG data. […]

This sustained effect [in the EEG] was not statistically reliable (i.e. no significant clusters were obtained). This could either be due to the lower signal-to-noise ratio of EEG or because EEG and MEG measure non-identical neural sources [30], which makes it possible that only one of the two methods captures a neural process of interest."

In sum, the sustained response seems to be a very weak response that does not correlate with behaviour.

We hope that our arguments above address this set of related concerns and make clear how our observation of sustained neural responses is nonetheless informative to the field.

6) I also have some concerns about the tACS results, which I find interesting. I have some trouble understanding why the tACS has to stop before the target to influence the target detection performance. Based on the MEG results, the sustained response is way weaker than the entrained response, which seems to predict a much stronger effect for "ongoing" tACS.

We thank the Reviewer for this comment which indeed raises an interesting question. We believe that there are some critical differences between the two experiments which can explain this result.

In both experiments, we use an oscillatory "echo" – sustained oscillations after the offset of stimulation – to draw conclusions about underlying mechanisms; i.e. demonstrating the neural and perceptual impact of endogenous oscillations entrained during *preceding* rhythmic stimulation. In both experiments, this echo provides us with a "clean" way of measuring endogenous oscillations entrained by a rhythmic stimulus. Measuring endogenous oscillations directly, i.e. *during* the rhythmic stimulus, is difficult as the stimulus evokes additional neural processes (e.g., evoked responses) which interfere with our ability to measure endogenous oscillations.

These neural processes, assumed to be active during stimulation, differ between the two experiments and might explain the discrepancy between results the Reviewer is referring to. In Experiment 1, given that rhythmic sounds were presented at a comfortable level, sensory stimulation produced strong and regular evoked responses. In contrast, the alternating current applied during tACS in Experiment 2 does not evoke neural activity. Instead, it leads to regular changes in the membrane potential of neurons which are assumed to entrain endogenous oscillations, but also produce rhythmic interference with speech processing in a way that does not necessarily involve endogenous oscillations (as the stimulation alternates between moments of strong stimulation, at the peaks and troughs, and no stimulation, at the zero crossings).

The difference in magnitude between rhythmic responses measured *during* and *after* rhythmic stimulation will depend on the strength of neural processes that occur – in addition to entrained endogenous oscillations – during but not after stimulation: In Experiment 1, evoked neural activity will clearly dominate responses measured during stimulation, leading to a pronounced response decrease after the offset of sensory stimulation. In Experiment 2, such a dominance by non-oscillatory processes was not expected, given the low intensity of the applied current. Our results are in line with this assumption, showing no statistically significant difference between the two tACS conditions (ongoing and pre-target).

The Reviewer rightly points out that, in Experiment 2, the phasic modulation of speech perception is (at least numerically) larger after than during stimulation. However, the two processes, assumed to operate during stimulation (e.g., rhythmic interference vs entrained endogenous oscillations during tACS) do not necessarily add up – indeed, they might even interfere with each other. This seems particularly likely if, as suggested by MEG findings, different neural sources are involved. Such an interference might lead to an overall rhythmic response that is reduced when compared to an effect that is due to endogenous oscillations alone (i.e. *after* tACS). In Experiment 1, this hypothetical interference might not play an important role during sensory stimulation, given evoked responses dominating entrained oscillations to a much larger degree.

In the revised manuscript, we now discuss these points in more detail (p. 21):

"In Experiment 2, the phasic modulation of speech perception observed after tACS (in the pre-target tACS condition) was not significantly different from that during tACS (in the ongoing tACS condition). In light of results from Experiment 1, where the sustained rhythmic response was clearly weaker than the entrained one, this might seem surprising. Importantly however, the process that interferes with our ability to measure endogenous oscillations during rhythmic stimulation is not identical in the two experiments. In Experiment 1, rhythmic sensory stimulation produced strong, regular evoked activity which dominates the response in the entrained time window. In Experiment 2, the current applied during tACS alternated regularly between periods of strong stimulation (at the tACS peaks and troughs) and no stimulation (at the zero crossings). This, according to our assumptions, might produce rhythmic modulation of speech perception that does not necessarily involve endogenous oscillations (perception might simply "follow" the amount of current injected). However, tACS is not strong enough to evoke neural activity [49,50], and the described effect will not dominate responses as strongly as sensory stimulation in Experiment 1. Moreover, such a phasic effect on speech perception does not necessarily combine additively with that produced by entrained endogenous oscillations – indeed, these two processes might even interfere with each other. Consequently, and in line with our results, rhythmic modulation of speech perception is not necessarily expected to be stronger when both processes interact

(regular changes in current vs entrained oscillations in the ongoing tACS condition) as compared to an effect that is due to endogenous oscillations alone (in the pre-target tACS condition)."

7) Furthermore, the tACS phase is correlated with the phase of the evoked EEG response, instead of the phase of the sustained EEG response. Nevertheless, the tACS effect is observed after the tACS stimulation. In other words, the tACS phase is likely to reflect the phase of the sustained response caused by tACS. Therefore, I have trouble understanding why the phase of sustained tACS response is correlated with the phase of entrained EEG response instead of the phase of the sustained EEG response.

This is an interesting question, and one which we can only answer speculatively. As explained above, EEG has a lower signal to noise ratio than MEG, and is sensitive to different orientations of underlying neural sources. These might be reasons why the sustained oscillatory response is not statistically reliable in the EEG. At the same time, electrical activity measured with EEG is more closely related to neural populations that are stimulated with tACS than with the neural populations measured with MEG – EEG and tACS will be similarly affected by current flow in the skull and other non-neural tissues. We therefore believe that EEG is the more appropriate neural measure to use for the analysis the Reviewer refers to, a point which we will address in detail below. We agree with the Reviewer that, given the results from Experiment 1, we would intuitively expect the phase of the sustained (and not entrained) EEG response to be predictive for the sustained tACS phase. However, such an effect might not be detectable (even if it exists) if the sustained EEG response *itself* is not reliable, for reasons explained above. The fact that the phase of the entrained EEG response predicts the sustained tACS response might indicate that the entrained EEG captures well the phase of endogenous oscillations, but this hypothesis requires additional testing. All of this is now discussed in the revised manuscript, along with a suggestion to use electrophysiological methods that are also closely related to tACS, but have a higher signal to noise ratio (p. 26):

"Perhaps surprisingly, given results from Experiment 1, the phase of the entrained, but not sustained EEG response was predictive for the phase of the sustained tACS effect. This result might be explained by the fact that, possibly due the lower signal to noise ratio of EEG, the sustained oscillatory response was not statistically reliable in the EEG in Experiment 1 (Fig. 5A). Consequently, a link between sustained oscillatory effects in EEG and tACS might not have been detectable, even if it exists, simply because the former was not measured reliably. Nevertheless, our finding that the entrained EEG response predicts sustained tACS phase indicates that entrained EEG responses can capture the phase of endogenous oscillations, despite observations of simultaneous evoked neural activity. MEG, showing statistically robust sustained responses (Fig. 2), is not as closely related to tACS as EEG (as its signal is not affected by the same distortions by bone and tissue) and is therefore less likely to be predictive of tACS outcomes (cf. Fig. S3). Future studies may need electrophysiological methods with higher signal to noise ratio than EEG, such as electrocorticography, ECoG, to test the relationship between sustained neural responses and tACS-induced changes in perception in more detail."

8) Additionally, the correlation between tACS phase and EEG phase is interesting, but I wonder why EEG instead of MEG is used in this analysis. MEG and EEG were simultaneously recorded. However, the MEG data were used to show the existence of a sustained response while the EEG data were used to correlate with the tACS phase. Please explain why the MEG and EEG data were used for different purposes.

We thank the Reviewer for this point, which prompted us to explain our rationale in more detail. As explained above, we focused our principal analyses of neural responses on MEG data, due to its higher signal to noise ratio. Note however, that we also provide corresponding results for EEG in Fig. 5A.
The current applied to the scalp during tACS is distorted by skull and tissue before it reaches the brain. The electrical signal produced by the brain is similarly distorted before being captured using EEG electrodes attached to the scalp. Importantly, MEG is not affected by such distortions. Consequently, EEG is methodologically closer to tACS than MEG, and is thus our method of choice when combining the two experiments. We have revised our manuscript to make this clearer (p. 13/14):

"Rather than the MEG data reported earlier, we analysed the concurrent EEG data collected during Experiment 1 and relate this to tACS effects observed in Experiment 2 in the same participants. This is because EEG is methodologically closer related to tACS than MEG: Both tACS and EEG, but not MEG, are similarly affected by distortions in current flow in the skull and other, non-neural tissues [29,30]. We therefore tested whether we can use EEG data to predict individual differences in φ_{tACS}."

Nevertheless, we have repeated our analysis combining the two experiments (Fig. 5), using MEG instead of EEG data (p. 40):

"Although methodologically more distant to tACS than EEG (only the latter two are affected by distortions by skull and tissue), we repeated the procedure for the simultaneously acquired MEG data (Fig. S3). Here, to avoid phase cancellation effects, z-values were calculated separately for each of the 204 gradiometers and then averaged across the two gradiometers in each pair, yielding one z-value for each of the 102 sensors positions (note that z-values from Rayleigh's test are always larger or equal to 0)."

The result is reproduced below and now included as Fig. S3. We were unable to reproduce the effect reported with EEG data, as expected given the reduced similarity between MEG and tACS (p. 16).

"As expected from its increased dissimilarity to tACS, MEG responses measured in Experiment 1 did not reveal any predictive value for tACS results from Experiment 2 (Fig. S3)."

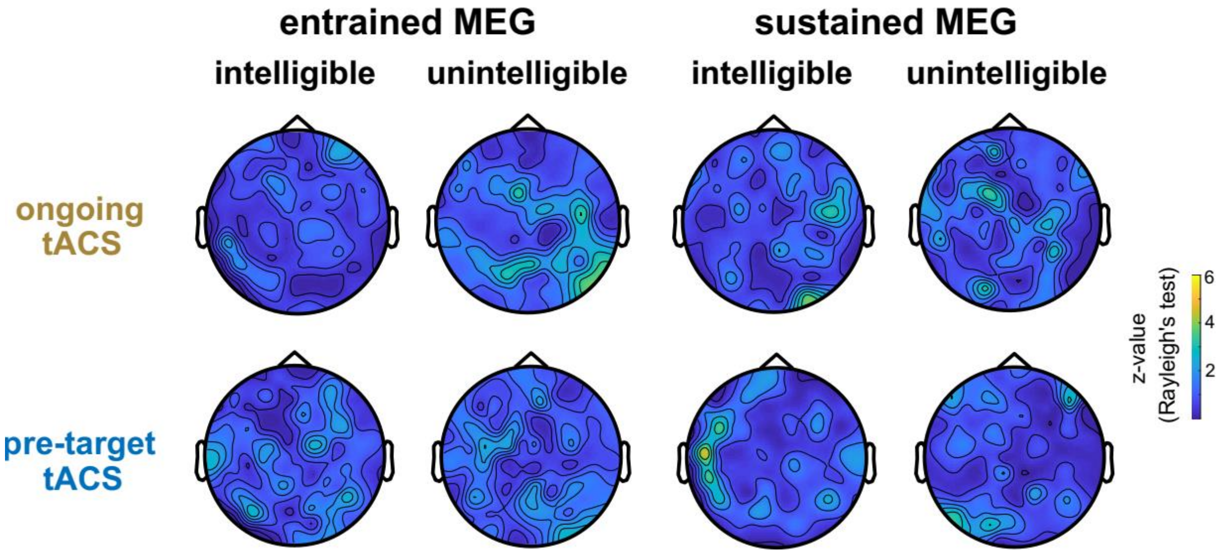

<u>Figure S3.</u> ***Using MEG responses to predict optimal tACS phase.*** *Same as Fig. 5D, but using MEG instead of EEG data from Experiment 1.*

Reviewer #2

The manuscript investigates whether neural entrainment to speech and to tACS sustains after the cessation of the stimulation. This observation would be key evidence that observed neural entrainment involves the recruitment of endogenous neural oscillations. For this, two experiments were performed with the same participants: one MEG/EEG experiment and one tACS experiment. The results show that the neural entrainment to intelligible speech outlasts the stimulus. The findings also show that tACS affects perception after the end of electrical stimulation, and that there is a correspondence between the EEG phase during entrainment and the tACS phase that leads to most accurate speech perception.

The study provides new important insights into the mechanisms underlying neural entrainment. It reports an extensive amount of data that are thoroughly analyzed. I find the main results convincing and particularly exciting. I still have a few questions mostly on the tACS results.

We thank the Reviewer for these positive comments and for interesting questions that helped us further improve the manuscript.

1) What I find most surprising in the tACS results is that the modulatory effect seems to be stronger in the "pre-target" tACS condition than in the "ongoing" TACS condition. I think this point needs to be more extensively discussed in the manuscript, especially with regards to past work on tACS-phase effects in auditory/ speech processing.

We thank the Reviewer for raising this point and note that a similar comment was offered by Reviewer 1. As before, we would like to bring to this Reviewer's attention that the phasic modulation of speech perception did not statistically differ between the two conditions and hence firm conclusions concerning the cause of this difference are without statistical support. Nevertheless, we agree with the Reviewer that it seems surprising that the phase effect is (at least numerically) larger after tACS (i.e. in the pre-target condition) rather than during tACS (in the ongoing condition). In the revised manuscript, we now discuss this finding in detail. We believe that it can be explained by the assumption that two different processes exist simultaneously while tACS is applied (rhythmic interference with perception vs entrained endogenous oscillations). These processes do not necessarily add up and might even interfere with each other. Such an interference might lead to an overall rhythmic response that is reduced when compared to an effect that is due to endogenous oscillations alone (i.e. *after* tACS). The relevant paragraph is copied below (p. 21 in version with tracked changes):

"In Experiment 2, the phasic modulation of speech perception observed after tACS (in the pre-target tACS condition) was not significantly different from that during tACS (in the ongoing tACS condition). In light of results from Experiment 1, where the sustained rhythmic response was clearly weaker than the entrained one, this might seem surprising. Importantly however, the process that interferes with our ability to measure endogenous oscillations during rhythmic stimulation is not identical in the two experiments. In Experiment 1, rhythmic sensory stimulation produced strong, regular evoked activity which dominates the response in the entrained time window. In Experiment 2, the current applied during tACS alternated regularly between periods of strong stimulation (at the tACS peaks and troughs) and no stimulation (at the zero crossings). This, according to our assumptions, might produce rhythmic modulation of speech perception that does not necessarily involve endogenous oscillations (perception might simply "follow" the amount of current injected). However, tACS is not strong enough to evoke neural activity [49,50], and the described effect will not dominate responses as strongly as sensory stimulation in Experiment 1. Moreover, such a phasic effect on speech perception does not necessarily combine additively with that produced by entrained endogenous oscillations – indeed, these two processes might even interfere with each other. Consequently, and in line with our results, rhythmic modulation of speech perception is not necessarily expected to be stronger when both processes interact (regular changes in current vs entrained oscillations in the ongoing tACS condition) as compared to an effect that is due to endogenous oscillations alone (in the pre-target tACS condition)."

We address the relation to previous work in the following point.

2) I am confused in particular by the discussion section, e.g. p. 659-660:"Our finding of enhanced speech perception therefore supports the hypothesis that tACS can enhance neural entrainment if it is applied in the absence of a "competing" entraining stimulus." I do not follow this argument. Ongoing tACS could potentially interfere with neural oscillations if presented at the wrong phase and/or frequency, but should in principle enhance oscillatory activity if presented at the correct phase? And based on the past literature (Riecke et al., 2018; Zoefel et al.,2018; 2020), I would still expect a phasic modulation of perception and/or neural response during the "ongoing" tACS condition. Do the authors have an explanation for this apparent discrepancy?

Again, we thank the Reviewer for this comment as it helped us clarify one point which was not sufficiently clear in the original version of our manuscript. The respective paragraph refers to our previous study (using the same electrode configuration; Zoefel et al 2020, *JOCN*) reporting that tACS disrupts speech perception rather than enhancing it. In that study, tACS was applied during the presentation of rhythmic speech; it is possible that the rhythmic sounds entrained oscillations up to the limit of what can be physiologically possible such that tACS could not enhance it further. In the current study, such a "competing" stimulus was absent. Our finding of enhanced speech perception by tACS is therefore in line with this assumption.

It is again important to note that we did not find a statistical difference in phasic modulation of speech perception between the two tACS conditions. Nevertheless, we agree with the Reviewer that the absence of a reliable phase effect in the ongoing tACS condition seems surprising, given previous work. We speculate that a reason for this might again be the absence of an additional entraining auditory stimulus. In our revised manuscript, we now provide a detailed discussion of these points and emphasize differences to previous studies (p. 26):

"It is of note that the phasic modulation of speech perception was not statistically reliable when the target was presented during tACS (i.e. in the ongoing tACS condition). This result seems in contrast to previous work [7–11]. However, in those studies, participants listened to and reported longer speech sequences while they were asked to detect a single target word (presented in background noise) in the current study. The quasi-regular rhythm of such sequences might act as an additional entraining stimulus which could boost or interact with tACS effects (see also next paragraph), in particular when perception is tested during tACS. Future studies should test the interesting question of whether and how the rhythmicity of the speech stimulus affects the efficacy of tACS during and after its application.

In previous work, using the same electrode configuration as applied in Experiment 2, we reported that tACS can only disrupt, and not enhance speech perception [8]. We previously hypothesized that this is because tACS was applied simultaneously with rhythmic speech sequences, which as Experiment 1 of our study showed can themselves entrain brain activity. If neural entrainment to the speech sequences were already at the limit of what is physiologically possible, tACS might only be able to disrupt, but not to enhance it further. Importantly, in the current study, tACS was applied during non-rhythmic background noise, i.e. without any simultaneously entraining stimulus. Our finding of enhanced speech perception therefore supports the hypothesis that tACS can enhance neural entrainment. However, if it is applied simultaneously with a strong "competing" entraining auditory stimulus, tACS might only be able to disrupt entrainment."

3) I think also that the conclusion l.337 that "rhythmic changes in speech perception outlast the period of tACS" seems a bit too strong with regards to the actual behavioral results (i.e. because of the absence of significant results in the "ongoing" tACS condition).

We appreciate the Reviewer's concern and changed the conclusion so that it does not depend on results from the ongoing tACS condition anymore (p. 13):

"Together, we found rhythmic changes in speech perception after the offset of tACS, which depend on the duration of the preceding stimulation. This finding demonstrates that tACS can induce rhythmic changes in neural activity that build up over time and continue beyond the period of stimulation. Both of these effects are consistent with endogenous neural oscillations being entrained by tACS."

We believe that this claim is justified, given the observed results (phasic modulation of speech perception and effect of tACS duration in the pre-target tACS condition), and hope that the Reviewer agrees with us.

4) Because there is no ramping on and off of the stimulation, could the participants feel when the current stopped in the trial? Could they guess they were in the "pre-target" condition, or in the "ongoing" condition?

This is an important point which we now discuss in the manuscript (p. 33):

"We verified in pre-tests that turning on or off the electric stimulation does not produce any sensation that is temporally so precise that participants can distinguish the two conditions (note that tACS is applied intermittently in both conditions, only with different timings relative to the target word). However, we did not measure potential sensations quantitatively during the experiment to avoid drawing attention to the transient nature of our tACS protocol. However, even if tACS sensations differed between the two conditions at the relevant time points (e.g., during target presentation), they seem unlikely to have affected the hypothesized phasic modulation of word report (for this to happen, participants would also need to distinguish different tACS phases, and relate these phases to the time at which the target is presented; see [8] for further discussion). Rather, we might expect a generic effect of tACS such as a difference in overall word report accuracy (averaged across phase). This result was not observed in the current study and hence we feel confident that the phasic effects of pre-target tACS are due to entrainment of underlying neural mechanisms."

5) Have the authors looked at the correlation between MEG phase and tACS data? I understand that EEG might reflect activity of networks most likely affected by the tACS current, but it could be interesting to correlate tACS and MEG data knowing that the sustained MEG response is more robust.

We thank the Reviewer for this suggestion, which we followed. As we explained to Reviewer 1, EEG is methodologically closer to tACS than MEG, and our method of choice when combining the two experiments. In addition to the analysis suggested by the Reviewer, we have revised our manuscript to make this clearer (p. 13/14):

"Rather than the MEG data reported earlier, we analysed the concurrent EEG data collected during Experiment 1 and relate this to tACS effects observed in Experiment 2 in the same participants. This is because EEG is methodologically closer related to tACS than MEG: Both tACS and EEG, but not MEG, are similarly affected by distortions in current flow in the skull and other, non-neural tissues [29,30]. We therefore tested whether we can use EEG data to predict individual differences in φ_{tACS}*."*

We have repeated our analysis combining the two experiments (Fig. 5), using MEG instead of EEG data (p. 40):

"Although methodologically more distant to tACS than EEG (only the latter two are affected by distortions by skull and tissue), we repeated the procedure for the simultaneously acquired MEG data (Fig. S3). Here, to avoid phase cancellation effects, z-values were calculated separately for each of the 204 gradiometers and then averaged across the two gradiometers in each pair, yielding one z-value for each of the 102 sensors positions (note that z-values from Rayleigh's test are always larger or equal to 0)."

The result is reproduced below and now included as Fig. S3. We were unable to reproduce the effect reported with EEG data, as expected given the reduced similarity between MEG and tACS (p. 16):

"As expected from its increased dissimilarity to tACS, MEG responses measured in Experiment 1 did not reveal any predictive value for tACS results from Experiment 2 (Fig. S3)."

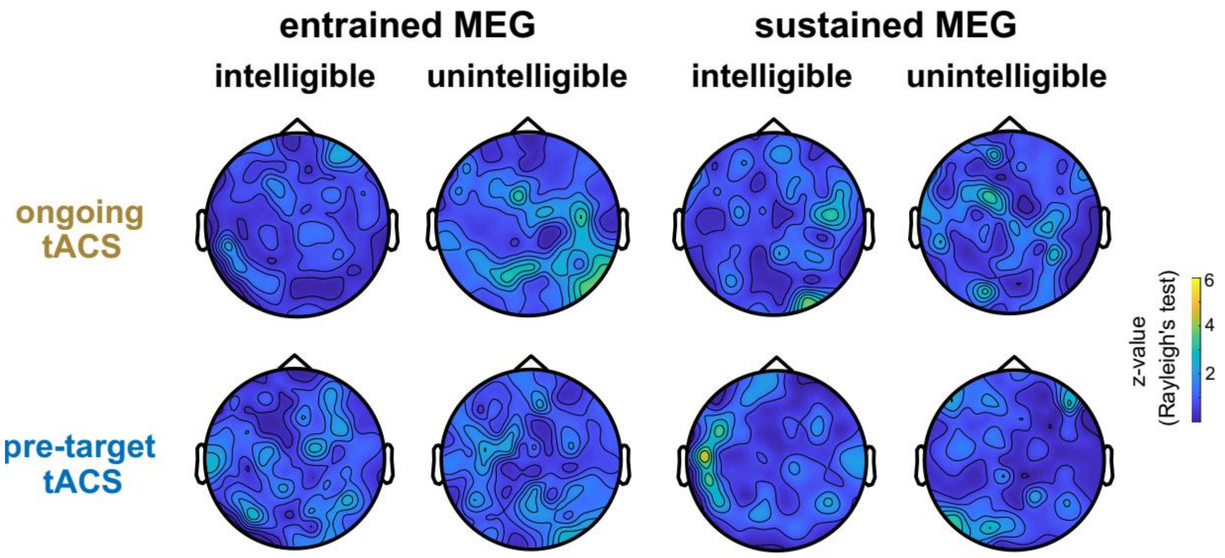

<u>Figure S3.</u> ***Using MEG responses to predict optimal tACS phase.*** *Same as Fig. 5D, but using MEG instead of EEG data from Experiment 1.*

6) Considering that sustained entrainment is not observed for unintelligible speech, would you consider that this phenomenon is restricted to speech processing? What about non-verbal rhythms, in auditory and other sensory modalities?

The finding of enhanced oscillatory processes for intelligible (as compared to unintelligible) speech is indeed common in the literature (e.g., Peelle et al. 2013, *Cereb Cortex*; Gross et al. 2013, *PLOS Biol*). However, we emphasize that our results do not necessarily mean that endogenous oscillations are restricted to the processing of speech: Sustained rhythmic responses to non-verbal stimuli have also been demonstrated, and neural oscillations play an important role for perception across modalities (e.g., Lakatos et al. 2013, *Neuron*; Spaak et al. 2014, *Curr Biol*). Speculatively, we propose several explanations for the observed dominance of intelligible speech to produce rhythmic neural activity, now included in our manuscript (p. 19):

"Our results should not be taken as evidence that endogenous neural oscillations are irrelevant for the processing of sounds other than human speech (e.g., see [40–42]). However, they might suggest that endogenous oscillations are optimized to process speech, due to its quasi-rhythmic properties [3,44]. Additionally, it is possible that the increased salience of intelligible speech (as compared to noise or tone stimuli) enhances participants' alertness and encourages higher-level processing, which has been shown to lead to enhanced oscillatory tracking of rhythmic structures [45,46]."

Reviewer #3

I am much in favour of this manuscript. It contains many important data points--obviously collected with great care and analysed by and large with impressive ingenuity--that there is a biophysical reality to neural entrainment and its behavioural corollaries, and that tACS, in a limited way, can perturb this behavioural (i.e., speech comprehension) outcome.

Not much of this study is per se truly new or unexpected, and the senior authors have published extensively on this--yet, given how contentious the claim of neural entrainment as a mechanism still is, a well-done study like this clearly deserves notice. The results demonstrated in Figure 5 to me are the single most important and most new element, but they also thus should raise most robustness checks (see below).

We thank the Reviewer for these positive comments, and for further suggestions to improve the manuscript.

1) It should also be noted that the Achilles heel of this study is the low N.
(In fact, I would not bet much money on the main result in Figure 5 (and in fact all correlations with behaviour here) replicating. This could most easily be settled, if it were simply -- replicated. Given that we are inmidst a pandemic, this is a problem I concur) I do think, however, the authors could do more to comfort their readers that the result in Figure 5 is solid: How much does it hinge on the (contentious; cf. Boateng et al.) individual peak-aligning? How much does it depend on a t-test with df=17 being used? How about comparing the entire two "patterns" in panels F,G instead? and so forth.

(Before we address the Reviewer's concern, we note that, due to the inclusion of an additional figure (described below), Figures 4 and 5 are now Figures 5 and 6, respectively. In our response, we use the new figure numbers.)

We agree with the Reviewer that – given their importance for the field – our results should be replicated in future work. Unfortunately, three out of four authors have left the place where this research was conducted. Together with the current unusual circumstances, as the Reviewer points out, this makes it difficult for us to provide further data at this time. However, as we will explain in detail below, we do believe that our finding is already robust and not subject to the concern about peak-aligning, raised by the Reviewer.

The most relevant results for our argument come from the comparison between EEG and tACS as shown in Fig. 5D (rather than Fig. 6, referred to by the Reviewer): Individual responses to tACS can be predicted from EEG responses to rhythmic intelligible speech. This analysis has been run without any pre-selection of conditions or sensors and is therefore unbiased – the obtained results are statistically robust (z-values of > 6) and apparent with stringent (FDR-)correction for multiple comparisons. The analysis does not involve any re-alignment either, as the phase difference between EEG and tACS is calculated at the level of individual participants (Fig. 5B,C; these differences are later subjected to a Rayleigh's test to obtained group-level statistics shown in Fig. 5D).
These results demonstrate that we can predict "best" and "worst" tACS phases for individual participants based on independent EEG data from the same participants. Given those results, it is a logical consequence that, if we re-align behaviour at these predicted phases, then speech perception at the predicted best (or worst) phase should be relatively (in)accurate – this is exactly what we observed in Fig. 6. The primary purpose of Fig. 6 was to illustrate the implications of results described earlier in the manuscript rather than to provide additional confirmation of our findings using peak-alignment methods. In the revised manuscript, we have made this clearer (p. 16 in version with tracked changes):

"Based on the consistent phase shift between $\varphi_{EEGvsSound}$ and φ_{tACS} shown in Figure 5E, however, it should be possible to predict optimal tACS phase for single participants from EEG responses aligned to rhythmic intelligible speech. We tested this prediction in an additional analysis, as illustrated in Fig.

6 (see also Materials and Methods). This analysis was designed to illustrate the implications of findings depicted in Fig. 5D for future applications (e.g., when optimising tACS methods for use in interventions), rather than for providing new results.

[…]

As intended, word report accuracy was highest at the predicted optimal phase lag (0 in Fig. 6F)."

And (p. 41):
"The primary purpose of this re-alignment is to illustrate implications of results obtained in the analysis described in the preceding paragraph (Fig. 5D)."

To conclude, results shown in Fig. 6 do *not* hinge on re-alignment and t-tests, as they illustrate results obtained without either of the two (Fig. 5D).

Fig. 6 addresses an additional, independent question. It is a common problem in tACS research that best phases for performance are not consistent across participants. Previous work has therefore aligned best phases at a common phase bin (e.g., Busch et al 2009, *J Neurosci*; Riecke et al 2018, *Curr Biol*; Zoefel et al 2018, *Curr Biol*) before data was analysed further. Performance in this bin is trivially a maximum and cannot be included in subsequent analyses or, as the Reviewer mentions, is prone to bias outcomes (Boateng et al). Consequently, it has been difficult to determine whether tACS enhances and/or disrupts speech perception (as opposed to "only" modulating it), as the phase bin with highest accuracy is "lost" during this alignment procedure (it cannot be determined whether accuracy in the bin used for alignment is high due to improved perception during tACS or merely due to the effect of cross-subject alignment).

We here used the fact that, while the best phase in the pre-target tACS condition could be predicted from EEG data, that in the ongoing tACS condition could not. Results from the latter condition can therefore be used as a "baseline": If tACS data is re-aligned *based on (non-predictive) EEG data*, then this condition should reflect outcomes under the null hypothesis of no tACS-induced modulation of performance. Of course, given results shown in Fig. 5D, it is no surprise that re-aligned data in the pre-target tACS condition (where we know already that EEG is predictive for tACS) is different from that in the ongoing tACS condition (i.e. the "baseline"). However, this final analysis showed us *how exactly* it is different: Is speech perception enhanced or disrupted (or both). Here, we chose a t-test for statistical comparison as it is optimally suited to answer this question – the relevant comparison concerns only one phase bin (pre-target vs ongoing condition at the predicted best or worst phase bin, respectively). In our view, it is unnecessary to compare all phase bins with each other (comparing "patterns" as the Reviewer suggested), as these are less relevant in this case. Finally, although data is re-aligned for this analysis, (1) this is done for both conditions (pre-target and ongoing), avoiding a specific bias, and (2) any such bias would not change the outcome in one of the directions tested (enhancement vs disruption of perception).

In the revised manuscript, we stated the following (p. 17):

"Given that entrained EEG is predictive for φ_{tACS} only in the pre-target tACS condition (Fig. 5D), there must be some phase bins in which accuracy differs between the two tACS conditions after EEG-based re-alignment. However, these previous analyses did not reveal the direction of this difference (enhancement vs disruption). We therefore compared performance at the predicted optimal tACS phase […]"

2) In sum, I very much welcome this study and would be happy for it to enter the canon. However, I urge the authors to bolster their case by an extensive series of clean-up in the domains of researcher degrees of freedom (see below) and statistical reporting/analysis. This will help make a much more solid case.would need to be convinced more of key take-homes. I outline a few more points in more detail below.

We thank the Reviewer for these suggestions which we hope to have followed satisfactorily – we provide details below.

***

3) My main problem for the overall conclusion is in fact one of robustness. ~N=20 is not much for any between-participants/generalising inference when it comes to predicting behaviour.
The researcher degrees of freedom in this paper have been immense obviously. The reader would thus need more demonstrations, or at the very least better rationale, to decide how robust key results are against these choices. A key point here is the switch from demonstrating a brain effect using MEG data in Exp 1, but using EEG data for the comparison with behavioural/tACS effects from Exp 2. Why was this done at all? Why should it not be possible to demonstrate the Fig. 5 effect in source space of MEG?

The Reviewer raises two important points – MEG vs EEG and sensor vs source space – which we address separately.

<u>MEG vs EEG</u>

We focused on MEG data to analyse data from Experiment 1, due to a higher signal to noise ratio as compared to EEG. However, we note that corresponding results for EEG were already included in the original submission (Fig. 5A).

The current applied to the scalp during tACS is distorted by skull and tissue before it reaches the brain. The electrical signal produced by the brain is similarly distorted before being measured using EEG at the scalp. Importantly, MEG is not affected by such distortions. Consequently, EEG is methodologically closer to tACS than MEG, and was a-priori our method of choice when combining the two experiments (Figs. 5 and 6). We have revised our manuscript to make this clearer (p. 13/14):

"Rather than the MEG data reported earlier, we analysed the concurrent EEG data collected during Experiment 1 and relate this to tACS effects observed in Experiment 2 in the same participants. This is because EEG is methodologically closer related to tACS than MEG: Both tACS and EEG, but not MEG, are similarly affected by distortions in current flow in the skull and other, non-neural tissues [29,30]. We therefore tested whether we can use EEG data to predict individual differences in φ_{tACS}."

Nevertheless, we have repeated our analysis combining the two experiments (Fig. 5), using MEG instead of EEG data (p. 40):

"Although methodologically more distant to tACS than EEG (only the latter two are affected by distortions by skull and tissue), we repeated the procedure for the simultaneously acquired MEG data (Fig. S3). Here, to avoid phase cancellation effects, z-values were calculated separately for each of the 204 gradiometers and then averaged across the two gradiometers in each pair, yielding one z-value for each of the 102 sensors positions (note that z-values from Rayleigh's test are always larger or equal to 0)."

The result is reproduced below and now included as Fig. S3. We were unable to reproduce the effect reported with EEG data, as expected given the reduced similarity between MEG and tACS (p. 16).

"As expected from its increased dissimilarity to tACS, MEG responses measured in Experiment 1 did not reveal any predictive value for tACS results from Experiment 2 (Fig. S3)."

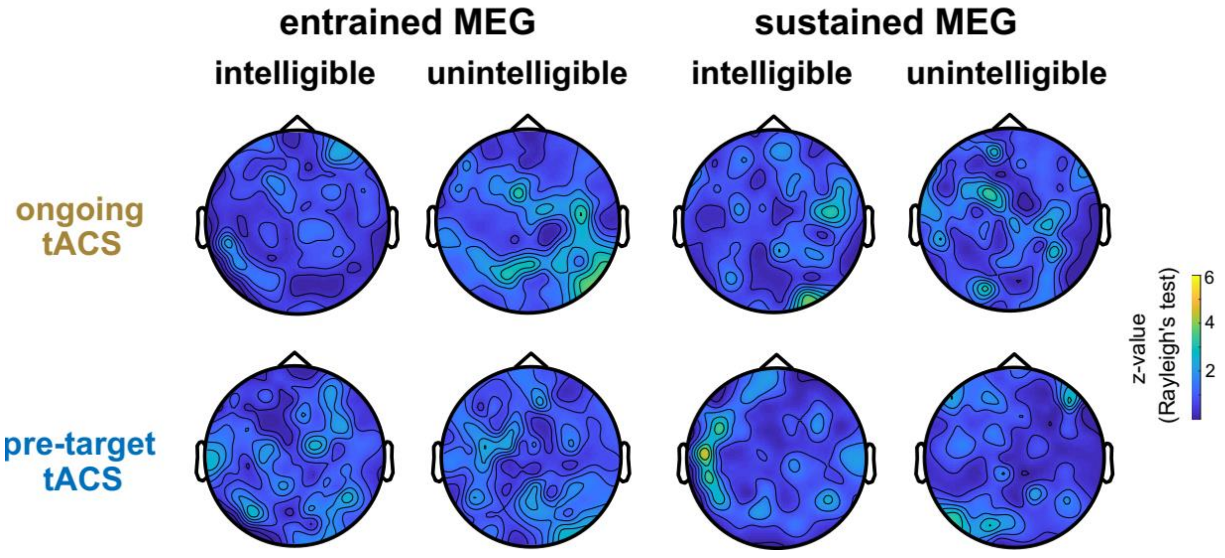

<u>Figure S3.</u> ***Using MEG responses to predict optimal tACS phase.*** *Same as Fig. 5D, but using MEG instead of EEG data from Experiment 1.*

<u>Sensor vs Source space</u>

In the current study, we use MEG to capture a signal that is expected to be relatively weak: A rhythmic brain response in the *absence* of rhythmic sensory stimulation. In such cases, analysing MEG data in source space is not necessarily superior to corresponding analyses in sensor space, since the signal to noise ratio is expected to be low (Jaiswal et al 2020, *Neuroimage*). For example, estimating the covariance matrix for LCMV beamforming is not straightforward without sensory stimulation. Similarly, it has been shown that phase-based connectivity measures are not reliable in source space when analysed in resting-state data (Colclough et al 2016, *Neuroimage*) (i.e. another situation without external input). While a sensor-space approach is assumption-free, reconstruction methods required for source space analyses make certain assumptions which are not necessarily valid and can lead to higher uncertainty in their outcome (Wendel et al 2009, *Comput Intell Neurosci*).
We emphasize that our study does not depend on making inferences about the exact spatial location or extent of neural effects. The spatial domain is therefore only of secondary importance for our results, and we prefer to use analytical approaches which make fewer assumptions – i.e. those in sensor space. We have clarified this point in the revised manuscript as follows (p. 37) and hope that the Reviewer agrees with our decision.

"MEG analyses in source space are not necessarily superior to those in sensor space, in particular when the signal of interest is expected to be relatively weak [76], such as in the current study (rhythmic brain responses in the absence of sensory stimulation). While sensor space analyses are assumption-free, reconstruction methods required for transformation to source space all make certain assumptions which can lead to increased uncertainty if they are invalid [77]. Given that we do not require inferences about the exact spatial location or extent of the hypothesized sustained oscillations, we focus here on analyses in sensor space. Nevertheless, we do also report results in source space for completeness, while emphasizing that they should be, for these reasons, be interpreted with caution."

4) A second point, pervasive throughout the ms., is the choice of sensors (see below) and time windows in statistical analysis.

We thank the Reviewer for this well-taken point of caution. Nevertheless, we insist that all of our main results have been obtained from a bias-free approach, i.e. without pre-selection of sensors or conditions: Fig. 2D,E shows sustained oscillatory responses in the MEG, including all sensors and conditions (of course after having restricted to pre-specified time windows without sensory stimulation). Fig. 4G (previously Fig. 3G) shows sustained rhythmic responses produced by tACS, including all conditions (i.e. stimulation durations). Fig. 5D (previously Fig. 4D) shows results for the combined experiments, including all sensors and conditions.

Only after having reported these main results, do we select sensors and conditions for follow-up analyses. But importantly, these analyses are not designed to test our main hypotheses – they further explore the effect, *given the reported main results*. In Fig. 3A,B, we demonstrate that the high RSR at the selected sensors is due to a high ITC at both stimulus rates; In Fig. 3C, we show the time course of the sustained response; In Fig. 5E, we show the time course of the correlation between EEG and tACS; In Fig. 6, we illustrate how our results can be applied. We therefore believe to have avoided the danger of double-dipping or circularity in our manuscript, and come back to this point below.

To address the Reviewer's concern, we have split the original Figure 2 into two figures: One which shows main results from Experiment 1 including all sensors and conditions for statistical analyses (now Figure 2), and another one exclusively showing follow-up analyses (Figure 3). We hope that this decision makes it easier for the reader to distinguish main from follow-up analyses.

In addition, we clarified in the revised manuscript (p. 8,9):

"All sensors and conditions were included in our main analyses (Fig. 2). We then explored the observed effects further (Fig. 3), restricting analyses of orthogonal contrasts to sensors which are most important for those main results."

[…]

"Although the presence of a sustained RSR is expected (given the method used to select the sensors), this result gives us valuable insight into the timing of the observed effect."

(p. 15)
"While main results are shown for all electrodes and conditions (Fig. 5D), we again restricted follow-up analyses to those which are most relevant, and based on orthogonal contrasts."

(p. 16)
"This analysis was designed to illustrate the implications of findings depicted in Fig. 5D for future applications (e.g., when optimising tACS methods for use in interventions), rather than for providing new results. We selected EEG data from the entrained time window and the EEG electrode (F3) which was most predictive for effects of pre-target tACS (Fig. 5D), and behavioural data from the same tACS condition. Such a selection is permitted as main results were already reported – without pre-selection – in Fig. 5D. For each participant i, we determined their individual $\varphi_{EEGvsSound}$ (Fig. 6B) and used it to estimate their individual φ_{tACS} (Fig. 6C), based on the difference between the two that was observed on the group level (Fig. 6A,C). Importantly, for the latter, data from participant i was excluded, avoiding circularity of the procedure."

5) -- Distributional properties of the new RSR index:
I am all for developing useful indices but demonstrating their theoretical and empirical properties in a supplemental figure is necessary.
Submitting the calculated RSR to an ANOVA sounds dangerous, to say the least. Both ITC and RSR should probably not be submitted to parametric analyses (mean differences, correlations, etc) at all. With the senior author's expertise in permutation statistics, I strongly recommend to make a consistent/overriding switch to such methods more suited to the data

We thank the Reviewer for these suggestions. Before we address the Reviewer's concern, we would like to briefly summarise why the new RSR index was constructed. We believe that its construction consists of appropriate analytical steps to remove potential bias or contamination, and the outcome is a clearer measure of whether or not sustained responses exist than could be provided by other standard measures in the literature. For example, inter-trial coherence (ITC) is, like other spectral measures, affected by aperiodic activity (showing a "1/f" profile), which can lead to larger ITC for lower frequencies, even in the absence of endogenous oscillatory activity. Moreover, other neural responses can bias ITC, such as the evoked response that can be produced by the omission of an expected stimulus (cf. Fig. 1E, which shows an increase in ITC after stimulus offset that is strongest at lower frequencies and visible for both stimulus rates). The RSR index contrasts ITC values observed at a given neural frequency during stimulation at a corresponding rate (e.g., 2-Hz ITC during 2-Hz speech), with ITC at the same neural frequency measured during stimulation with a different rate (e.g., 2-Hz ITC during 3-Hz speech). Consequently, generic processes that are independent of stimulus rate but that might affect ITC, such as an evoked response to stimulus omission, are removed. We have tried to clarify this point in the revised manuscript (p. 6):

"Spectral measures such as ITC can be biased by other neural activity than endogenous oscillations: For example, a response caused by the omission of an expected stimulus might produce an increase in ITC that is most pronounced at low frequencies (~250 ms in Fig. 1E). By contrasting ITC between two rate conditions, RSR removes such contamination if it is independent of stimulus rate (i.e. present in both rate conditions)."

A similar concern underlies permutation tests, suggested by the Reviewer: The surrogate distribution, constructed during these tests, is assumed to include all properties of the original data *except for* the hypothesized rhythmic response. However, this assumption does not necessarily hold in our dataset: Randomising trials or conditions might also abolish increases in ITC due to omission response or 1/f components, and hence lead to false positive effects. To test whether this is the case, we constructed a surrogate distribution by adding a random value to the phase extracted for each trial before re-calculating ITC values as described in the original manuscript. As shown below, ITC in the surrogate distribution is indeed not affected by the 1/f component that is visible in the original data. A more appropriate null distribution would however contain such a 1/f component.

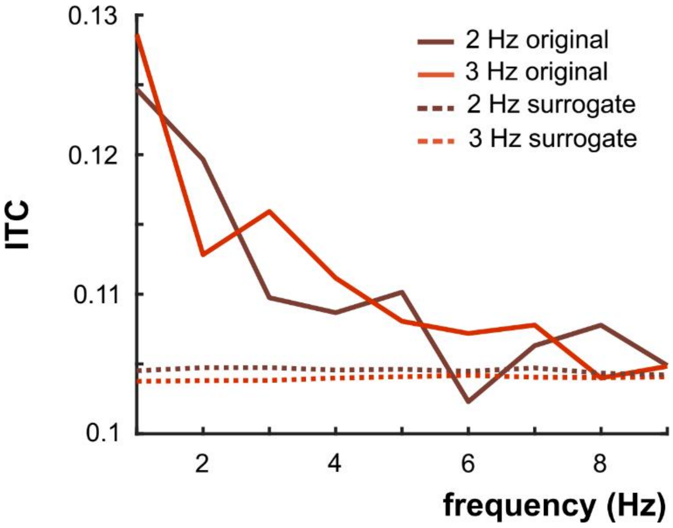

Figure R1. ITC as a function of neural frequency in the sustained time window, in response to the two stimulus rates (reproducing Fig. 3B), and for a surrogate dataset (mean across permutations; dashed lines). The latter was constructed by adding a value to the phase in each trial before calculating ITC. This procedure should abolish any phase consistency across trials and therefore simulate the null hypothesis of no rhythmic sustained response; however, it also abolishes the 1/f component visible in the original dataset.

We therefore believe that a comparison between two conditions (i.e. two stimulus rates) – i.e. the RSR – leads to statistically more robust results than corresponding permutation tests (p. 6):

"By contrasting ITC between two rate conditions, RSR removes such contamination if it is independent of stimulus rate (i.e. present in both rate conditions). This property makes it – in the present case – also superior to other commonly used approaches, such as permutation tests [21,22], which would not only

abolish the hypothesized rhythmic responses, but also non-rhythmic responses which produce high ITC for other reasons (e.g., evoked response to stimulus omission).”

[…]

(p. 9):
“In the sustained time window, subtracting 1/f components (dashed lines in Fig. 3B) from the data (continuous lines) revealed clearer peaks that correspond to the stimulation rate (or its harmonics). We note again the RSR discards such 1/f components by contrasting ITC values at the same two frequencies across the two stimulation rates.”

We ran additional verifications/analyses to satisfy the Reviewer’s concerns: First, we verified that our RSR index is indeed normally distributed. Second, we used the surrogate distribution, described above, to verify that the null hypothesis indeed corresponds to an RSR of 0. We found that the 95% confidence interval is centred on 0 and included this result in the revised manuscript (p. 35):

“For all sensors and conditions (intelligibility, duration) separately, we verified that the RSR is normally distributed ($p > 0.05$ in Kolmogorov-Smirnov test), a pre-requisite for subjecting it to parametric statistical tests. Note that such a behaviour is expected, given the central limit theorem (combining multiple measures leads to a variable that tends to be normally distributed). Fig. S5A shows the distribution of RSR, averaged across sensors and conditions. Finally, we constructed a surrogate distribution to verify that an RSR of 0 indeed corresponds to our null hypothesis. This was done by adding a random value to the phase in each trial before re-calculating ITC and RSR as described above, and repeating the procedure 100 times to obtain a simulated distribution of RSR values in the absence of a rhythmic response. This distribution of RSR values was indeed centred on 0, and its 95% confidence interval included 0 (Fig. S5B). Once again, this justifies our use of parametric statistical tests to confirm whether the observed RSR is greater than zero.”

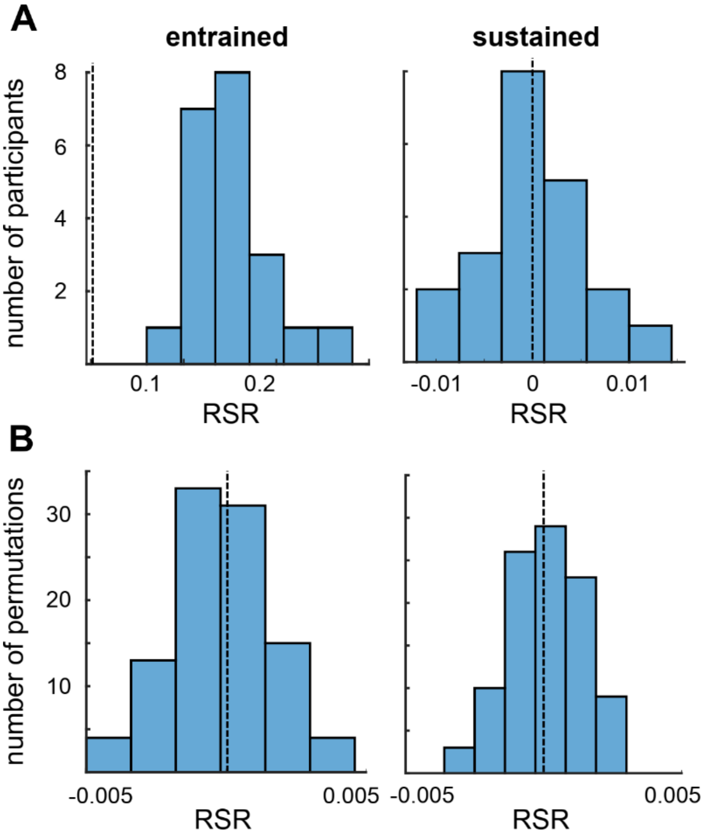

Figure S5. ***Control analyses validating RSR as an appropriate measure to reveal rate-specific rhythmic brain responses. A****. Distribution of RSR over participants. Note the approximate normal distribution as required for parametric tests (e.g., t-test against 0).* ***B****. Distribution of RSR, averaged across participants, in a surrogate dataset (see Materials and Methods). RSR is centred on 0 (dashed lines), validating our null hypothesis of RSR = 0. For all results shown here, RSR values have been averaged across sensors and conditions (corresponding to the average RSR shown in Fig. 2A,D), including those for which the RSR is not reliably different from 0. Statistically significant rate-specific responses after intelligible speech are shown in Fig. 2F. Note that x-axes are not identical across panels.*

6) Line 216 f: Selecting the sensors with the largest response runs an unnecessary danger of double-dipping/circularity. Your paper and your point in case will be much stronger if you present a principled way of analysing these data. In fact, I strongly suggest to present these analyses entirely in source space. Source space if the obvious forte of MEG. The prime advantage here would not only lie in a higher (if modest) degree of neurofunctional organisation specificity). More importantly, the move to source space in MEG acts as a spatial filter that can should improve SNR and benefit inference at al levels.

We hope that we have addressed both issues raised here (double dipping/circularity and source space analysis) in our previous response (points 1, 3, 4). For this reason we only briefly summarise our response here.
First, all main analyses (depicted in Figure 2) were run without pre-selection and included all sensors and conditions. Only orthogonal, follow-up analyses (listed in response to point 4), which explore effects further *given main results*, selected those sensors which seemed most relevant.
Second, we believe that MEG analyses in source space are not always superior to those in sensor space. This is true in particular if the signal of interest is expected to be relatively weak, and the spatial component of the results is only of secondary importance. We therefore decided to report all primary results in sensor space and hope the Reviewer agrees with our decision.

7) Line 228: My point about which sensors/signals to pick, the entrained vs sustained response correlations w/ behaviour deserve more attention. I am not sure whether the authors here want to run with a dissociation interpretation? Then the correlations would need to be tested against each other (unlikely to provide evidence for a difference, in fact; z test for paired correlations would only be significant if we assume a very high correlation between RSR from the two time windows). If the goal here is to argue for an absent interaction, though, predicting behaviour from both time windows in a regression model might be an option, showing that both contribute. In all cases, I was unclear what the authors want to convey with this (low-N, between-subjects, and thus notoriously unstable) correlation.

We thank the Reviewer for pointing out this issue – we fully agree that our report of correlations between RSR and behaviour required more detailed consideration.

The experimental task (detecting irregularities in the stimulus rhythm) was designed only to keep participants alert and attentive to the stimulus rhythm. We avoided direct behavioural measures of speech perception in Experiment 1 – despite this being a more relevant task for our main hypotheses – as speech perception (e.g., word report) tasks would only be possible for one condition (16-channel speech), but not the other condition (1-channel speech, which is completely unintelligible). We have motivated this decision more explicitly in the revised manuscript.
Given that the behavioural data is only of secondary importance in Experiment 1 and the statistical issues raised by the Reviewer (very high correlations between the two RSR would be required for follow-up analyses), we have decided to move the corresponding figures to Supporting Information, and to reduce the prominence of those results in the manuscript. We now explain these points as follows (p. 9/10):

"We did not measure the success of speech perception in Experiment 1. This is because such a task would have biased participants to attend differently to stimuli in intelligible conditions, making comparisons with neural responses in our unintelligible control condition difficult. Similarly, we refrained from using tasks which might have biased our measurement of endogenous oscillations in the silent period. For example, tasks in which participants are asked to explicitly predict an upcoming stimulus might have encouraged them to imagine or tap along with the rhythm. Our irregularity detection task was therefore primarily designed to ensure that participants remain alert and focused and not to provide behavioural relevance of our hypothesized sustained neural effect. Nevertheless, we correlated the RSR in both time windows (and at the selected sensors) with performance in the irregularity detection task (Fig. S2). […]"

8) line 314f: Again, I was unclear about the rationale of the authors: Is there or is there not a tACS x condition interaction, i.e. what can we conclude about the specificity of the tACS condition/window effect on word accuracy? The three separate z tests also suffer from lower sensitivity due to less data being included, of course.

Although we report a phasic modulation of speech perception that increases with tACS duration in the pre-target tACS condition, we do not find a significant interaction between tACS condition (ongoing vs pre-target) and tACS duration. As the Reviewer points out, this might be due to lower statistical reliability, caused by the increased number of conditions that leads to having less data available. As the interaction is not statistically reliable, we agree with the Reviewer that certain claims – in particular, that an effect of tACS duration is apparent *only* in the pre-target tACS condition – are not justified. In our revised manuscript, all claims that refer to this finding are therefore phrased carefully:

(p. 13):
[…] we found rhythmic changes in speech perception after the offset of tACS, which depend on the duration of the preceding stimulation. […] these effects are consistent with endogenous neural oscillations being entrained by tACS."

(p. 22):
"Our finding that tACS effects on perception increase with stimulation duration (Fig. 4G) is therefore clearly in line with oscillatory models. […] Although effects of tACS duration on behaviour were numerically larger and only statistically reliable in this [pre-target tACS] condition, we hesitate to conclude that this effect is specific to pre-target tACS since the condition by duration interaction was not reliable. […]"

Importantly, however, the lack of statistical difference between tACS conditions does not undermine our conclusions that true endogenous oscillations are entrained by tACS. These conclusions depend solely on observing phasic modulation and effects of duration in the pre-target condition which were statistically reliable throughout.

Minor:
9) reporting only p-values for cluster tests, correlation tests, etc is not state of the art.

We thank the Reviewer for pointing this out. More details have been included for these tests.
In addition, we now provide a link to data and analysis scripts:

https://osf.io/xw8c4/

We have also replaced results from the ANOVA (Fig. 2B,E) with repeated-measures t-tests that assess the same main effects and interactions. This was done to take into account the direction of the statistical outcome when comparing RSR across conditions. For example, RSR can be higher or lower for intelligible vs unintelligible speech, and this direction cannot be determined from the F-values obtained during an ANOVA and reported previously (other figure panels in Fig. 2 were unaffected by this issue as they test RSR against 0 using t-tests). Given equivalence between t-tests and ANOVA ($t^2 = F$) in experimental designs with two conditions per factor, this change does not affect statistical significance for individual sensors. However, it could alter cluster-based statistics if topographies contain relatively high t-values for positive and negative polarities in close proximity. This was not the case for the effect of intelligibility, and results therefore remain unchanged. However, the analysis revealed additional small effects of sequence duration for the entrained time window, and this is now described in the manuscript (p. 7):

"We also found a main effect of duration, revealing a preference for shorter sequences for left frontal sensors (cluster-based correction, $p = 0.02$; summed $t = -11.11$; 4 sensors in cluster) and one for longer sequences for parietal sensors (cluster-based correction, $p = 0.05$; summed $t = 6.83$; 3 sensors in cluster)."

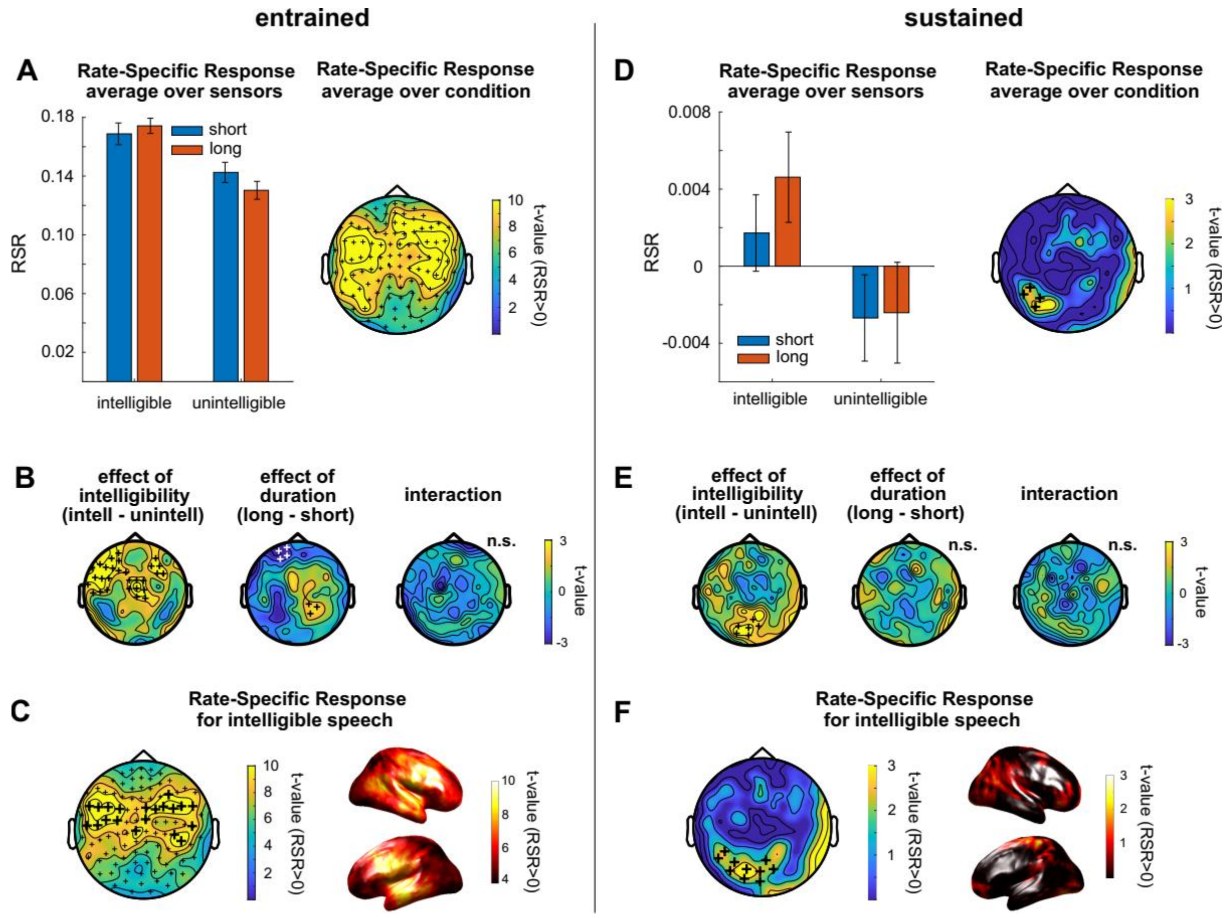

Figure 2. Main results from Experiment 1. A-C. *Results in the entrained time window. Bars in panel* ***A*** *show RSR in the different conditions, averaged across gradiometers and participants. Error bars show SEM, corrected for within-subject comparison. The topography shows t-values for the comparison with 0, separately for the 102 gradiometer pairs, and after RSR was averaged across conditions. Topographies in* ***B*** *contrast RSR across conditions. Topography and source plots in* ***C*** *show t-values for the comparison with 0 in the intelligible conditions. In all topographic plots, plus signs indicate the spatial extent of significant clusters from cluster-based permutation tests (see Materials and Methods). In* ***B****, white plus signs indicate a cluster with negative polarity (i.e. negative t-values) for the respective contrast. In* ***A*** *and* ***C****, this cluster includes all gradiometers (small plus signs). In* ***C****, larger plus signs show the 20 sensors with the highest RSR, selected for subsequent analyses (Fig. 3).* ***D-F****. Same as A-C, but for the sustained time window.*

10) font size in all but a few figure panels is prohibitively small

Font size has been increased for all figure panels.

11) line 424: Just another illustration of my major point of contention, where the EEG channel choice seems overly tailored to the data. More efforts of choosing sensors/ROIs/time windows independent of the data to be tested on it need to be entertained by the authors.

We briefly summarise our response from above (points 1, 4), where this issue was discussed in detail. We insist that there is no circularity in the paper. Our main analyses include all channels and conditions; only then, *given the effect* already revealed in the initial analyses, are these findings followed up by

analyses which explore orthogonal contrasts in the data in the most relevant sensors and conditions. The paragraph the Reviewer refers to is an example of such a follow-up analysis (now Fig. 6), which includes channels that have been selected from main results reported earlier in the paper (now Fig. 5D).

12) Subheading "Sustained oscillations produced by tACS enhance, but do not disrupt speech perception" does need statistical qualification (i.e. interaction pattern). The authors run danger once more of arguing with not necessarily meaningful differences of differences.

We maintain that such a statement is justified, given results reported in our original submission. This statement is a consequence of two independent statistical approaches by which we test two independent hypotheses (1: tACS enhances perception; 2: tACS disrupts perception). We first test whether word report accuracy at the predicted optimal tACS phase in the pre-target tACS condition is better than “baseline” accuracy (i.e. in the ongoing tACS condition as explained above). We then repeat the procedure for the predicted worst tACS phase. In these two tests, we find enhanced speech perception at the predicted best tACS phase, but no disrupted speech perception at the predicted worst tACS phase, leading to the statement referred to by the Reviewer. We hope that with this justification that this review now finds this text acceptable.
