# Supplementary material for: Sustained neural rhythms reveal endogenous oscillations supporting speech perception
Source: PLoS Biol. 2021 Feb 26;19(2):e3001142. doi: 10.1371/journal.pbio.3001142 (PMC7946281; doi:10.1371/journal.pbio.3001142)
Supplement: Attachment — Submitted filename: rebuttal_FINAL.pdf [file pbio.3001142.s008.pdf]

We thank all editors and reviewers for a very constructive review process which has clearly improved our manuscript. Below we address the remaining concerns and hope that our manuscript can now be considered acceptable for publication.

While preparing data for publication, we noticed that two conditions (2s and 3s) were swapped during the analysis of behavioural results from Experiment 1 (Fig. 1B). When corrected, our results revealed that irregularities were easier to detect in shorter sequences, not in longer ones as previously reported. All statistical (F- and p-)values that were previously reported are correct, only the two conditions were interchanged. As these behavioural data were only secondary to the overall aim of the study, this mistake did not affect any of the other analyses or results reported. All data provided was thoroughly checked and verified once again. We apologize for this mistake.

Editorial comments

--Please indicate whether your protocol, approved by the Cambridge psychology research ethics committee adhered to the Declaration of Helsinki or other national or international ethical guidelines.

This information has been added (the study was carried out in accordance with the Declaration of Helsinki).

-- Please include the ID number of the protocol approved by the Cambridge Psychology Research Ethics Committee.

The application number has been included.

-- Please note whether the informed consent obtained from participants in this study was oral or written. If consent was oral, please explain why

This information has been added (the informed consent was written).

--Please provide (or indicate where in the manuscript this can be found), as a supporting file, a spreadsheet that contains the individual numerical values that were used to generate the summary statistics show in Figure 1B-F; 2A-F; 3A-C; 4D,E-G; 5A-D; 6A-G; Figure S1A-D; S2A-B; S3; S4; S5A-B. For an example spreadsheet see here:
http://www.plosbiology.org/article/info%3Adoi%2F10.1371%2Fjournal.pbio.1001908#s5

Please also indicate, within each figure legend, where this underlying data may be found and ensure your supplemental data file/s has a legend.

The data spreadsheet has been provided for figure panels showing summary statistics (note that some of the panels listed only show example data, e.g. from individual participants), and figure legends have been updated accordingly.

-- Please include in your deposition in OSF a README file that would allow the reader to link your data files to each of the figures displaying quantitative data, by explaining how the data was analyzed to generate the final plots and graphs.

The readme file has been updated accordingly.

--Please ensure that your Data Statement in the submission system accurately describes where your data can be found.

This has been done.

Reviewer 1

The authors have done a good job revising the manuscript. However, I still have a few concerns.

We thank the Reviewer for these positive remarks and hope that we were able to satisfy remaining concerns with our changes in the manuscript and replies below.

1. "Like other spectral measures, ITC is affected by aperiodic ("1/f") activity, leading to larger ITC for lower frequencies without necessarily involving endogenous oscillatory activity."

ITC certainly shouldn't be affected by the 1/f spectrum. The 1/f shape is for the power spectrum while the ITC analyzes the phase coherence over trials. In fact, the authors should explain why the ITC reported here has a 1/f trend.

We agree with the Reviewer that ITC is a measure that is independent of power and therefore should – at least theoretically – not be affected by aperiodic activity (i.e. by higher power for lower frequencies). In practice, however, phase estimates are more reliable for a signal with higher power, due to the increase in signal-to-noise ratio. We confirmed this assumption in a simple simulation. We designed a periodic signal of interest (2-Hz sine function with a fixed phase). In 1000 simulated trials, we added some noise to the signal and then extracted ITC from these 1000 trials. We systematically varied the amplitude of the signal, while the amplitude of the noise was fixed (leading to a higher signal-to-noise ratio for larger signal amplitudes). As expected, ITC increased with signal amplitude as shown in the graph below.

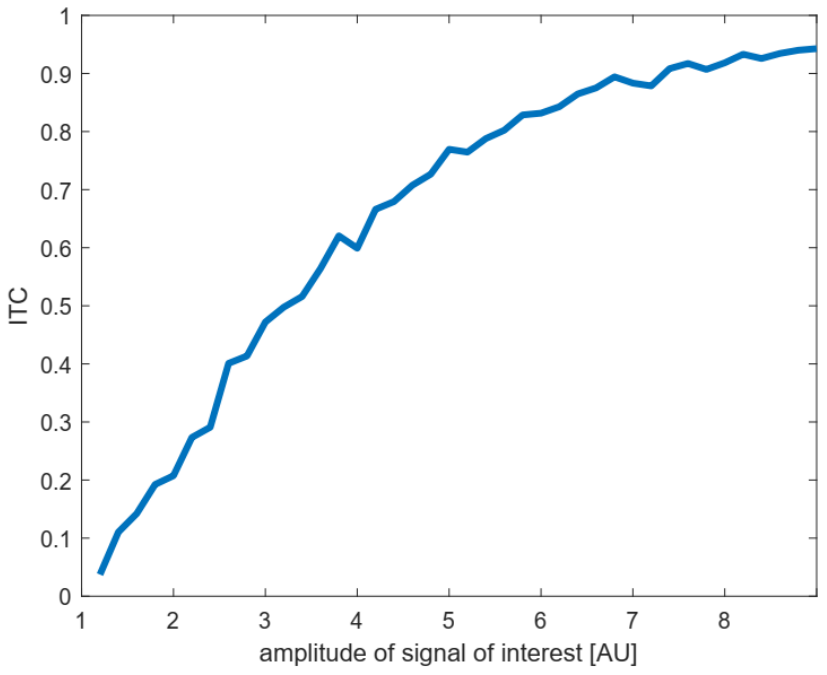

It is therefore conceivable that the 1/f shape, observed for ITC in our data, is an indirect effect of aperiodic activity that leads to higher power and more reliable phases at lower frequencies. This is now mentioned in our revised manuscript:

"These 1/f components can bias the outcome of spectral analyses [23,24]. Although this primarily affects estimates of oscillatory power (e.g., higher power for lower frequencies), higher power leads to more reliable estimates of phase and therefore potentially also to higher ITC (even though this measure is analytically independent of power, see above)."

2. "The current applied to the scalp during tACS is distorted by skull and tissue before it reaches the brain. The electrical signal produced by the brain is similarly distorted before being captured using EEG

electrodes attached to the scalp. Importantly, MEG is not affected by such distortions. Consequently, EEG is methodologically closer to tACS than MEG."

I can't agree with this statement. By this logic, the authors should have stimulated the bilateral auditory cortices by applying tACS at the position of Cz, instead of applying tACS bilaterally. If a signal is conducted from position A to position B, there is no garantee that stimulation on position B will activate position A, and vice versa.

We thank the Reviewer for insisting on this point. We now provide additional references to back up the claim that the relationship between measured voltages in EEG and currents applied with tACS is closer than the equivalent relationship between magnetic flux measured with MEG and tACS (30-32 below).

We agree with the Reviewer that, given the reciprocity between EEG and tACS, applying tACS at Cz should target those neural sources which produce EEG responses at Cz (though only if other tACS electrodes could be placed at scalp locations showing opposite responses, i.e. polarity). However, we had no a-priori hypothesis about the topographical distribution reflecting entrained endogenous oscillations (activity at Cz might only reflect evoked auditory responses), and therefore we decided to use a tACS configuration that has reliably modulated speech perception in previous similar studies (Zoefel et al 2020, *JOCN*). Nevertheless, in line with the Reviewer's argument, the simplest interpretation of the reciprocity between EEG and tACS predicts that EEG activity at an electrode position that corresponds to our tACS configuration (T7/T8) should correlate most strongly with our tACS effects. Our results indicate that such a simple interpretation does not hold, and that more complex mechanisms underlie our effect. In addition, it remains a possibility that tACS at those EEG electrode positions showing maximal predictive values for tACS effects would lead to even stronger effects. This is now discussed in our manuscript:
"According to the simplest interpretation of the reciprocity between EEG and tACS, if the signal from a neural source is captured at a certain (EEG) electrode position, then the same electrode position should be efficient in stimulating this neural source (with tACS) [30–32]. Vice versa, if a tACS electrode configuration is successful in targeting a certain neural source, then activity from this source should be measurable with EEG at this electrode position. As the topographical pattern of EEG signals with high predictive value for tACS (fronto-occipital pattern; Fig. 5D) was different from the tACS electrode position (T7/8), our results indicate that this simple interpretation does not hold and that more complex mechanisms underlie our observations. This could be because multiple neural sources are involved and interact to produce the topographical distribution measured with EEG, while the tACS protocol used can only reach one or some of them. It is also possible that tACS modulates the efficacy of sensory input to activate neural ensembles, while EEG measures the output of these ensembles. Differences in neural populations contributing to input vs output processing, including their orientation to the scalp, might explain the observed deviance from simple reciprocity between EEG and tACS. Finally, it is possible that even stronger modulation of perception could be achieved if tACS were applied at those (fronto-occipital) EEG electrode positions showing maximal predictive values for tACS effects – this could be explored in future work."

30. Helmholtz H. Ueber einige Gesetze der Vertheilung elektrischer Ströme in körperlichen Leitern mit Anwendung auf die thierisch-elektrischen Versuche. Ann Phys. 1853;165: 211–233. doi:10.1002/andp.18531650603

31. Dmochowski JP, Koessler L, Norcia AM, Bikson M, Parra LC. Optimal use of EEG recordings to target active brain areas with transcranial electrical stimulation. NeuroImage. 2017;157: 69–80. doi:10.1016/j.neuroimage.2017.05.059

32. Fernández-Corazza M, Turovets S, Luu P, Anderson E, Tucker D. Transcranial Electrical Neuromodulation Based on the Reciprocity Principle. Front Psychiatry. 2016;7: 87. doi:10.3389/fpsyt.2016.00087

Finally, I still have some concerns about the hypothesis that any neural activity that can last for a couple of seconds reflects endogenous activity. After a hit, a bell can ring for several seconds but I'm not sure if this kind of ringing should be called endogenous.

We thank the Reviewer for raising this interesting point. The pitch which a typical bell (to follow the Reviewer's example) produces, does not depend on how hard or often it is struck, but on its intrinsic properties (such as its size). Even the ringing of a bell can therefore be described as being constrained by an endogenous (internally determined) process. Such a process, however, seems too rigid to be used by the brain, which needs to be flexible enough to adapt to input properties (e.g. stimulus rate) to a certain extent (such that neural oscillations are observed within a specific frequency range). This is why we believe that our demonstration of frequency-specific oscillations is important since it provides evidence for such a flexible neural mechanism.

As we measured neural or behavioural output from the brain, by definition, our acquired data will include some components that are endogenous to the brain. We use the term "endogenous oscillation" not to describe a process that is *exclusively* endogenous, but rather to refer to the presumed origin of the *rhythmic component* in the neural signal. If the neural process operates rhythmically even in the absence of a rhythmic stimulus, then we can conclude that it has an intrinsic rhythm, qualifying the process as an endogenous oscillation. On the other hand, a series of evoked neural responses would reflect a process that is endogenous but not intrinsically rhythmic – rather, the rhythmic component in the signal arises from the rhythm of the exogenous stimulus.

Nevertheless, as the Reviewer implies, it is of course still possible that the sustained oscillations observed are generated by a passive process, i.e. they need an exogenous stimulus to operate. However, even in this case it would still reflect an endogenous neural process. This question – whether endogenous oscillations reflect a passive or active mechanism – is already discussed in detail in the manuscript (cf. Fig. 7). For example (Introduction):

It is possible that such a[n entrainment] process entails a passive, "bottom-up" component during which oscillations are rhythmically "pushed" by the stimulus, similar to the regular swing of a pendulum (that is, the endogenous oscillation is "triggered" by an exogenous stimulus). On the other hand (and not mutually exclusive), an active, "top-down" component could adjust neural activity so that it is optimally aligned with a predicted stimulus. Importantly, in both cases we would anticipate that oscillatory brain responses are sustained for some time after the offset of stimulation: This could be because predictions about upcoming rhythmic input are upheld, and/or neural oscillations are self-sustaining and (much like a pendulum swing) will continue after the cessation of a driving input.

In the revised Introduction, we now explicitly mention the possibility that endogenous oscillations could be triggered by an exogenous stimulus (in green).

In the Discussion, we dedicated a whole section to this question (Rhythmic entrainment echoes – active predictions or passive after-effect?). We hope that this convinces the Reviewer that we are aware of, and have sufficiently addressed, this important point.
